# GSA Launches G3: Genes | Genomes | Genetics

**DOI:** 10.1534/g3.111.000414

**Published:** 2011-06-01

**Authors:** Brenda J. Andrews, Paul W. Sternberg, Tracey DePellegrin Connelly

**Affiliations:** * Founding Editor-in-Chief, G3: Genes | Genomes | Genetics;; † President, Genetics Society of America, and; ‡ Executive Editor, GSA Journals

We are proud to present the inaugural issue of G3: Genes | Genomes | Genetics, an open-access journal published by the Genetics Society of America (GSA). The journal’s team of over 60 associate editors and 4 section editors, all practicing scientists—your peers—have come together to form a new, open-access journal with a unique mission and vision. The Editorial Board of G3 taps the expertise of the community of geneticists in the widest sense, from microbes to humans, from individuals to populations, and from classic “wet lab” experimentation to the most recent innovations in bioinformatics.

Geneticists have never been more prolific, and there is no shortage of venues for presenting their discoveries. So why do we need yet another scholarly journal? We see two compelling reasons.

First, because new sequencing and other technologies have greatly expanded the experimental reach of geneticists, we are now able to apply genetic analysis to species previously out of our range. Many of these and other studies may not be of wide interest or provide a significant mechanistic insight (yet), and thus are not appropriate for the broad audience of the GSA’s long-standing, flagship journal GENETICS. We and the GSA Board of Directors saw the need for a journal to serve the genetics community by providing an outlet for dissemination of findings and experimental resources in genetics and genomics—an outlet unrestricted by subjective editorial criteria of perceived significance or predicted breadth of interest. In brief, we are interested in publishing papers that describe useful, well-executed, and lucidly-interpreted genetic studies of all kinds.

We recognize our responsibility to enable documentation of reagents and resources, description of foundational work in developing areas of genetics, and datasets for meta-analysis, to name a few. We see a need for a journal to provide a unified home for reporting genome sequences, genetic and physical maps of organisms, mutant screens, QTL mapping, and many other important and useful datasets. We also recognize the need to provide fair and rapid peer review and be a respected source of interesting papers—a place where people want to publish their work. We’ve launched G3 to fulfill these needs.

Second, publication of data, ideas, and conclusions is the bedrock of science. We believe that practicing scientists must play a central role in that process. Many journals will publish our work, but few carry the imprimatur of an organization of peers with a long history of supporting our field. The Genetics Society of America has an illustrious record in scientific publishing with its groundbreaking journal GENETICS, the first American journal of genetics, established in 1916 by the founders of our field. G3, as a sister journal of GENETICS, will continue the tradition of science run by scientists.

G3 will maintain a standard of quality to make the journal desirable, both for publication and for perusal. The data will be high-quality and the conclusions well-supported. The journal is focused on publishing results of genetic research—research that encompasses a range of experimental analyses in established model systems, as well as investigations into less well-trodden genetic territory. G3’s focus on genetics means that the editorial board can remain relatively small and respond to the needs of our community.

The best journals offer innovative ways to present your work. For example, G3 (and GENETICS) provides links [within articles] to model organism databases, so with one click a reader can access a gene name and a wealth of associated information. In addition, we’ve responded to our community by providing a forum where all data are available—whereby researchers can drill deeper in their exploration for meaning. Beyond publishing research that meets our standards, G3 leaves it up to the community to determine its level of significance and utility. It’s often impossible to predict whether an article will have widespread importance. This is the serendipity and joy of scientific discovery.

And one of the best aspects of G3? After investing your resources and yourself in your research, you want a quick decision and prompt publication. G3 strives to offer a decision within 30 days of submission, and online publication within 45 days. G3 is fully open access, with a Creative Commons license that allows the freest use of the data: anyone can download, analyze, mine, and reuse the data provided that the authors of the article receive credit. We believe that rapid dissemination of useful data is the necessary foundation for analysis that leads to mechanistic insights. Our hope is that this strategy will spawn new discovery. And because the GSA journals have taken a leading role in ensuring full availability of all data related to an article, we pay more than just lip service to the meaning of open access.

Submit your work for publication in the newest journal of the GSA and trust decisions made by your peers. Because your research is important to you, it’s important to us.

